# The role of upfront lenalidomide maintenance for primary central nervous system lymphoma following first‐line methotrexate treatment: A retrospective study

**DOI:** 10.1002/cam4.7193

**Published:** 2024-05-13

**Authors:** Yan Zhang, Wei Wang, Danqing Zhao, Wei Chong, Chao Chen, Wei Zhang, Daobin Zhou

**Affiliations:** ^1^ Department of Hematology, Chinese Academy of Medical Sciences & Peking Union Medical College Peking Union Medical College Hospital Beijing China

**Keywords:** frontline maintenance, lenalidomide, methotrexate‐based induction chemotherapy, primary central nervous system lymphoma

## Abstract

**Background:**

Consolidation therapy improves the duration of response among patients with primary central nervous system lymphoma (PCNSL). Lenalidomide maintenance has shown encouraging results in older patients with PCNSL. Herein, we performed a retrospective, single‐center analysis to evaluate the effect of lenalidomide maintenance on the duration of response in patients with newly‐diagnosed PCNSL.

**Methods:**

Sixty‐nine adult patients with PCNSL who achieved complete remission or partial remission (PR) after induction therapy were enrolled. The median age of patients was 58.0 years. The maintenance group (*n* = 35) received oral lenalidomide (25 mg/day) for 21 days, every 28 days for 24 months; the observation group did not undergo any further treatment.

**Results:**

After a median follow‐up of 32.6 months, the maintenance group experienced fewer relapse events. However, the median progression‐free survival (PFS) was similar between groups (36.1 vs. 30.6 months; hazard ratio, 0.78; 95% confidence interval, 0.446). Lenalidomide maintenance significantly improved PFS and overall survival (OS) only among patients who experienced PR after induction. The median duration of lenalidomide maintenance was 18 months; lenalidomide was well tolerated and minimally impacted the quality of life.

**Conclusions:**

The present study was the first to evaluate lenalidomide maintenance as a frontline treatment among patients with PCNSL, PFS and OS did not improve, although the safety profile was satisfactory.

## 1 INTRODUCTION

Primary central nervous system lymphoma (PCNSL), a rare and distinct entity from diffuse large B‐cell lymphoma (DLBCL), accounts for 4% of newly‐diagnosed brain tumors. Currently, the treatment of young patients with PCNSL includes two phases: induction and consolidation.^1^ High‐dose methotrexate (MTX)‐based chemotherapy is the backbone of PCNSL treatment.^2^, ^3^, ^4^ There are two strategies during the consolidation phase: high‐dose chemotherapy plus autologous stem cell (ASCT) transplantation with thiotepa‐based conditioning and whole‐brain radiation therapy (WBRT).^5^, ^6^, ^7^ Three randomized controlled trials have demonstrated substantial benefits in terms of overall survival (OS) and progression‐free survival (PFS) in patients with newly‐diagnosed myeloma who received LEN maintenance following autologous stem cell transplantation (ASCT), in comparison to those in the placebo or observation group.^8^ However, maintenance is an alternative option in specific situations.

Lenalidomide (LEN), a second‐generation immunomodulatory drug with pleiotropic antitumor effects, has gained significant usage among patients with myeloma and DLBCL. LEN maintenance was initially introduced to the treatment of multiple myeloma.^9^ Several studies have evaluated the efficacy of LEN maintenance among patients with DLBCL. In 2017, the REMARC study showed that LEN maintenance for 24 months after obtaining a response substantially prolonged PFS in older patients with newly‐diagnosed DLBCL.^10^ LEN can effectively penetrate the blood–brain barrier (BBB) and affords moderate anti‐lymphoma activity in patients with PCNSL.^11^ Therefore, LEN is a good option for PCNSL as a maintenance treatment. In 2019, Vu et al.^12^ reported that low‐dose LEN maintenance was associated with excellent PFS and OS in older patients with PCNSL, as determined by a small‐scale retrospective study. Accordingly, the LEN maintenance strategy in patients with newly‐diagnosed PCNSL is worth investigating. In the current retrospective study, we aimed to evaluate the efficacy and safety of LEN maintenance in patients with PCNSL who responded to MTX‐based induction chemotherapy.

## 2 PATIENTS AND METHODS

### 2.1 Patients

This was a retrospective, single‐institution study. Immunocompetent adult patients with newly‐diagnosed PCNSL were enrolled at the Peking Union Medical College Hospital from 2003 to 2021, including some patients from a prospective study (REMLA trial; Rituximab, lEnalidomide Methotrexate induction tandem Lenalidomide mAintenance; registration number: 04120350). We included (1) patients treated with high‐dose MTX‐based regimens and (2) those who achieved complete remission (CR) or partial remission (PR) after induction therapy.

The demographic data and clinical features of all patients, including sex, age, Eastern Cooperative Oncology Group performance status, lactate dehydrogenase level, cerebrospinal fluid (CSF) cytology analysis, and brain magnetic resonance imaging (MRI) findings, were collected from medical records. In addition, we estimated the risk stratification of patients according to the International Extranodal Lymphoma Study Group (IELSG) score.

This retrospective analysis was performed in accordance with the protocol approved by the Peking Union Medical College Hospital Institutional Review Board. All patients provided informed consent.

### 2.2 Treatments and outcomes

All patients received a high‐dose MTX (HD‐MTX)‐based regimen. Several regimens were applied in this study: (a) HD‐MTX ± R regimen: MTX with or without rituximab 375 mg/m^2^; (b) R‐MA: rituximab methotrexate and cytarabine; (c) R^2^‐MTX: rituximab, LEN, and MTX; and (d) AB ± R alternative regimen: MTX, ifosfamide, vindesine, dexamethasone, carmustine, and teniposide, with or without rituximab, which was preferred before 2010 at our center; the corresponding details are described in Table S1 and our previous report.^13^ For patients with CSF involvement, a triple intrathecal injection therapy consisting of methotrexate, cytarabine, and dexamethasone was administered. The patients with intraocular involvement received a combined treatment approach involving intravitreal injection of methotrexate at a dose of 500 μg. After induction treatment, the responses were evaluated using contrast‐enhanced MRI, and all patients achieved CR or PR in our study.

In the LEN maintenance group, LEN was initiated within 6 weeks of the last induction cycle. LEN was administered at a starting dose of 25 mg/day from day 1 to 21 during a 28‐day cycle for 24 months until the completion of maintenance treatment, disease progression or relapse, and unacceptable toxicity. A dose‐reduction schedule was applied according to toxicity. There were three levels of LEN doses: level 1, 25 mg/day from day 1 to 21; level 2, 25 mg every 2 days from day 1 to 21; and level 3, 25 mg every 3 days from day 1 to 21. During maintenance therapy, tumor response assessment was performed clinically every 3 months with contrast‐enhanced MRI. Patients were evaluated annually at the end of maintenance therapy or at the time of treatment discontinuation. In the observation group, patients did not receive any further treatments until PCNSL relapse, and the follow‐up schedule was consistent with the LEN maintenance group.

The effects of treatments on quality of life (QoL) were assessed using the European Organization for Research and Treatment of Cancer QLQ‐C30 (EORTC QLQ‐C30 V3, simple Chinese edition) for patients from the prospective REMLA trial. These tests were performed before induction treatment, at the end of treatment (4 months), and at 6 months every 3 or 6 months thereafter. The questionnaires were conducted by experienced physicians. If patients discontinued LEN administration owing to toxicity or disease progression, the QoL evaluations were simultaneously discontinued.

Follow‐up data were obtained from patient records or by telephone. OS was assessed from the date of the first treatment until the date of the last follow‐up or death. PFS was calculated from the date of first treatment to the date of progression, relapse, death, or last contact.

### 2.3 Statistical analysis

The clinical characteristics and safety profiles of patients are summarized using descriptive statistics, and categorical variables are summarized using frequency tabulations. PFS and OS were estimated using the Kaplan–Meier test with 95% confidence intervals (95% CIs). The effects of baseline characteristics on PFS and OS were evaluated using log‐rank tests and Cox proportional hazards regression. All statistical analyses were performed using GraphPad Prism 7 (GraphPad Software Inc., San Diego, CA, USA) and SPSS version 25 (IBM Corp., Armonk, NY, USA).

## 3 RESULTS

### 3.1 Patients

Herein, we identified 69 eligible patients, with a median age of 58 (range, 17–78) years at the time of diagnosis, and 50.7% (*n* = 35) of patients were male. In total, 10 (14.5%) patients showed intraocular involvement. Only two patients did not undergo lumber puncture for CSF analysis at diagnosis. Nine (13.0%) patients had CSF involvement, as determined by cytology and/or flow cytometry.

Overall, 40 patients had sufficient pathological information to clarify the cell of origin (COO) subtype, 10 belonged to the GCB subtype, and 30 belonged to the non‐GCB subtype.

According to the IELSG prognostic model, 19 (27.5%), 35 (50.7%), and 13 (18.8%) patients were classified into the low‐, intermediate‐, and high‐risk groups, respectively. All patients completed induction therapy, and HD‐MTX ± R (*n* = 25) was the most common regimen, followed by the R^2^‐MTX (*n* = 18) and A + B alternative regimens (*n* = 17). Only one patient received the R‐MA regimen. Fifty‐seven (82.6%) patients achieved CR, and 12 (18.4%) achieved PR. The baseline clinical characteristics between the LEN maintenance and observation groups were similar (Table 1). However, there were between‐group differences in induction regimens. At our center, the A + B alternative regimen was the standard induction regimen from 2000 to 2010, whereas LEN maintenance was utilized since 2015, concomitant with the decreased use of WBRT. Nevertheless, both groups had similar remission status (CR rate 85.7% vs. 79.4%, *p* = 0.540).

**TABLE 1:** Demographic and clinical characteristics at baseline.

	LEN group (*n* = 35)	Observation group (*n* = 34)	*p*‐Value
Age (median, range)	60 (28–76)	56.5 (17–78)	0.172
Male (*n*, %)	15 (42.8%)	20 (58.8%)	0.232
IELSG risk group (*n*, %)			
Low	12 (35.3%)	7 (20.6%)	0.524
Intermediate	17 (48.6%)	18 (52.9%)	
High	6 (17.1%)	7 (20.6%)	
NA	0 (0%)	2 (5.9%)	
Treatment regimen (*n*)			
HD‐MTX ± R	10 (28.6%)	15 (44.1%)	<0.0001
R‐MA	1 (2.9%)	0 (0%)	
A + B	0 (0%)	17 (50%)	
R^2^‐MTX	24 (68.6%)	2 (5.9%)	
Autologous stem cell transplantation consolidation	2 (5.6%)	2 (6.0%)	>0.99
Whole brain radiation consolidation	2 (5.6%)	11 (33.3%)	0.004
Response after induction			
CR	30 (85.7%)	27 (79.4%)	0.540
PR	5 (14.3%)	7 (20.6%)	
Outcomes			
Relapsing	15 (42.9%)	23 (67.7%)	0.0385
Death	4 (11.4%)	18 (51.9%)	0.0002
Disease progression	3 (8.6%)	11 (32.4%)	
No relapsing death	1 (2.9%)	7 (20.6%)	

Abbreviations: AB, methotrexate ifosfamide vindesine dexamethasone carmustine and teniposide regimen; CR, complete remission; HD‐MTX ± R, methotrexate with or without rituximab; IELSG, International Extranodal Lymphoma Study Group; LEN, lenalidomide; PR, partial remission; R^2^‐MTX, rituximab lenalidomide and methotrexate; R‐MA, rituximab methotrexate and cytarabine.

### 3.2 Treatments exposure

Thirty‐five patients received LEN maintenance, and the median duration of LEN administration was 18 (range, 2–36) months. Approximately 14 (40.0%) patients completed maintenance, and 12 (34.2%) patients remained on maintenance at the time of the last follow‐up. Nine (25.7%) patients prematurely discontinued treatment, eight stopped due to disease progression, and only one stopped therapy following LEN‐related toxicities. However, 10 (28.6%) patients had at least one dose reduction during the maintenance; eight were reduced to level 2, and two were reduced to level 3.

### 3.3 Efficacy

**FIGURE 1:**
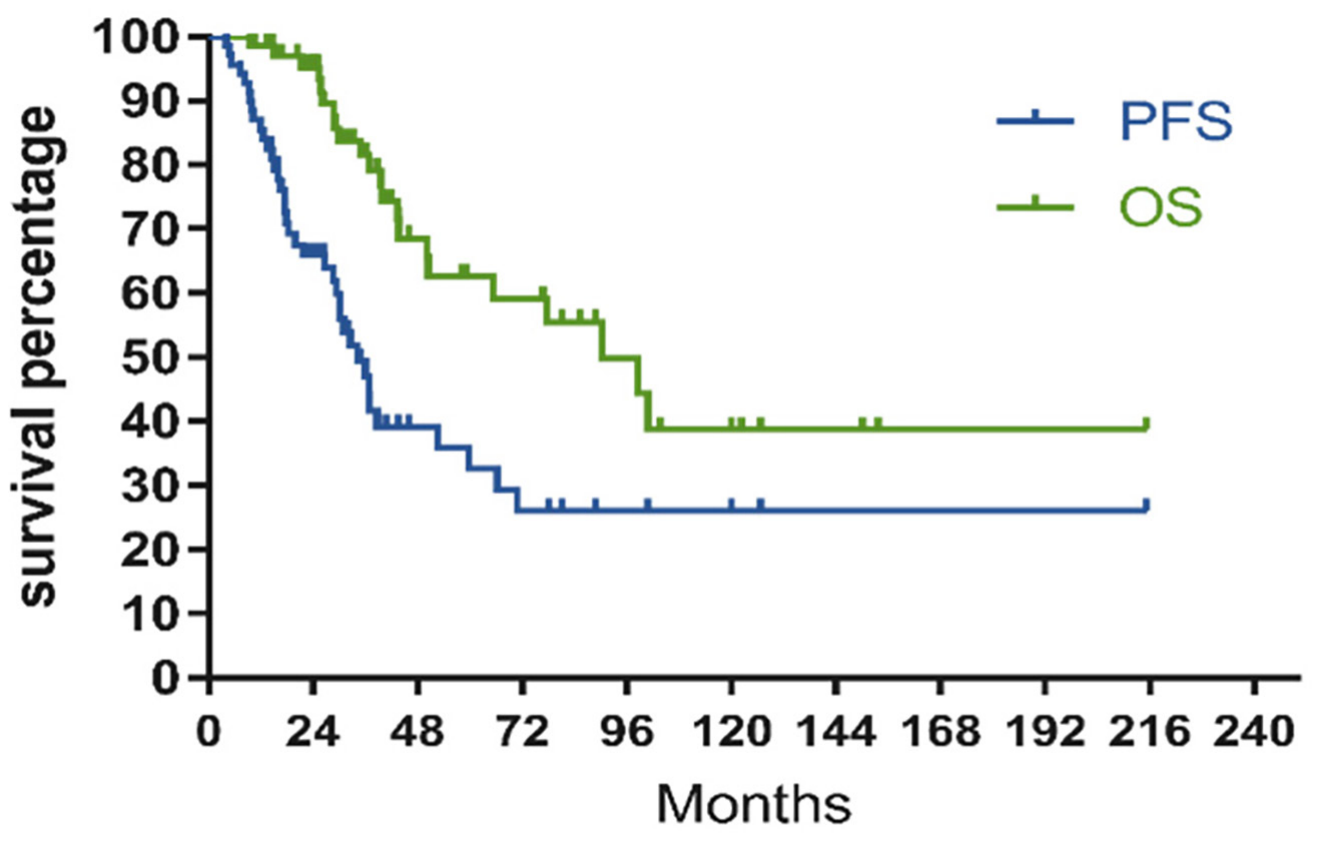
Kaplan–Meier curve of PFS and OS for the whole series. OS, overall survival; PFS, progression free survival.

The cutoff date was August 1, 2022, and the median follow‐up period was 32.6 (range, 10–85) months for the whole series. The LEN maintenance strategy was initiated in 2015 at our center, and the median follow‐up time differed between the LEN maintenance and observation groups (32.6 and 44.9 months, respectively). Thirty‐eight patients relapsed, and 47 were alive at the time of analysis. Fourteen patients died from disease progression, and eight died from other complications related to PCNSL and/or salvage treatment. Considering the whole series, the median PFS and OS were 34.1 months (95% CI, 26.4–41.8) and unreached (95% CI, 56.4–124.0 months), respectively (Figure 1). The median relapse times were 17.5 and 18.2 months in the LEN maintenance and observation groups, respectively, and the relapse rates at 2 years were similar (50.0% vs. 58.5%, *p* = 0.748).

**FIGURE 2:**
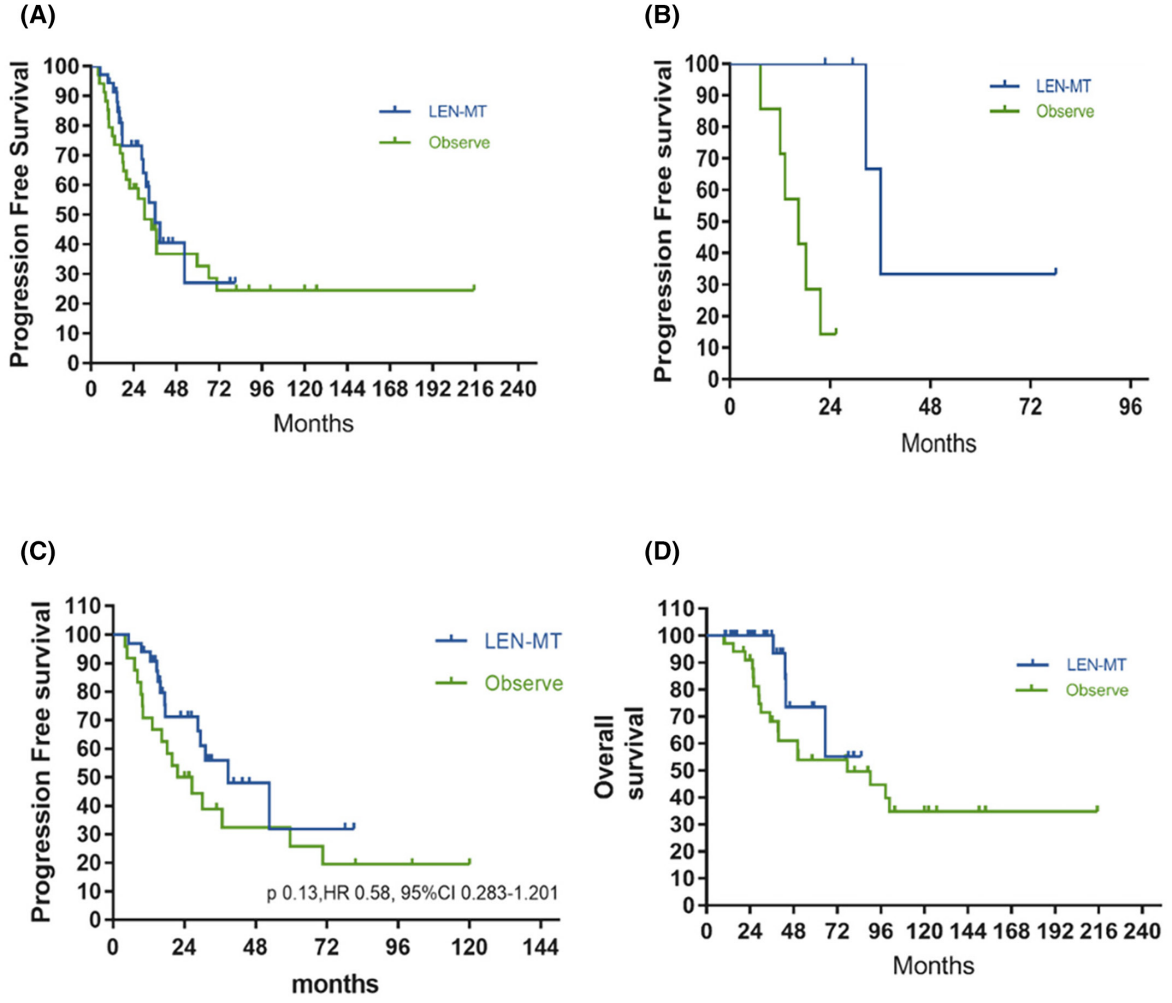
Survival of patients between LEN maintenance and observation groups. (A) Progression‐free survival. (B) PFS in patients who achieved PR. (C) PFS in patients without WBRT. (D) Overall survival. LEN, lenalidomide; MT, maintenance; OS, overall survival; PFS, progression free survival; PR, partial remission; WBRT, whole brain radiation therapy.

The median PFS was 36.1 and 30.6 months in the LEN maintenance and observation groups, respectively, indicating the absence of a significant difference in PFS rates between groups (Figure 2A; *p* = 0.446; hazard ratio [HR], 0.78; 95% CI, 0.412–1.479; long‐rank test). In the subgroup analysis, patients who achieved PR after induction therapy benefited from LEN maintenance, and the median PFS was prolonged from 16.4 to 36.1 months (Figure 2B; *p* = 0.0062; HR 0.17; 95% CI, 0.040–0.746; long‐rank test); however, the PFS among patients who achieved CR was not significantly prolonged after LEN maintenance (*p* = 0.964; HR, 0.98; 95% CI, 0.470–2.06; long‐rank test). In the subgroup analysis of WBRT status, patients who did not receive WBRT consolidation demonstrated a trend of PFS improvement in the LEN maintenance group (Figure 2C; 38.7 vs. 24.2 months; *p* = 0.13; HR, 0.58; 95% CI, 0.283–1.201; long‐rank test). Patients in the LEN maintenance group tended to have a longer median OS than those in the observation group (Figure 2D; unreached vs. 77.5 months; *p* = 0.091; HR, 0.4263; 95% CI, 0.177–1.028; log‐rank test). Furthermore, patients who achieved PR experienced a more significant benefit than those who achieved CR (Figure S1; 65.4 vs. 26 months; *p* = 0.063; HR, 0.28; 95% CI, 0.077–1.046; long‐rank test); the difference was almost negligible among patients with CR (*p* = 0.293; HR, 0.48; 95% CI, 0.141–1.603; long‐rank test). The LEN maintenance group did not demonstrate notable improvement in PFS or OS, considering demographic and disease characteristics at diagnosis, including age <60 years, COO, IELSG risk scores, and induction regimens.

We assessed the baseline cerebrospinal fluid (CSF) interleukin‐10 (IL‐10) levels in 25 patients from the lenalidomide maintenance group. No statistically significant difference was observed between patients who experienced relapse and those who did not (199.9 ± 96.98, *n* = 12 vs 234.7 ± 109.7, *n* = 13, *p* = 0.81, Student's *t*‐test).

The univariate analysis showed that the PFS and OS of all patients did not differ significantly according to the clinical characteristics at diagnosis (sex, age, COO, and IELSG high‐risk group), induction regimens, and consolidation treatment (Table 2). The CR status after induction therapy was associated with longer OS but not improved PFS.

**TABLE 2:** Univariate analysis of progression‐free survival and overall survival.

	PFS		OS	
	*p* Value	HR (95% CI)	*p* Value	HR (95% CI)
Male	0.344	1.36 (0.72–2.60)	0.930	0.96 (0.41–2.23)
Age >60	0.335	0.72 (0.37–1.40)	0.067	2.20 (0.95–5.11)
Non‐GCB	0.274	1.89 (0.60–5.92)	0.993	1.01 (0.09–12.08)
LEN maintenance	0.448	0.77 (0.40–1.50)	0.102	0.40 (0.13–1.20)
Induction regimen	0.834		0.154	
HD‐MTX ± R	–	–	–	–
A + B regimen	0.828	0.92 (0.43–1.98)	0.722	0.86 (0.35–2.07)
R^2^‐MTX	0.201	0.83 (0.37–1.89)	0.1040	0.23 (0.04–1.35)
IELSG risk	0.738		0.6301	
Low risk	–	–	–	–
Intermediate risk	0.469	0.76 (0.35–1.66)	0.787	1.16 (0.41–3.24)
High risk	0.566	0.75 (0.29–1.96)	0.352	1.78 (0.49–6.49)
Complete response	0.109	0.538 (0.25–1.15)	<0.001	0.203 (0.08–0.50)

Abbreviations: A + B, methotrexate ifosfamide vindesine dexamethasone carmustine and teniposide regimen; CR, complete remission; HD‐MTX ± R, methotrexate with or without rituximab; HR, hazard ratio; IELSG, International Extranodal Lymphoma Study Group; non‐GCB, non germinal center b‐cell; OS, overall survival; PFS, progression free survival; PR, partial remission; R^2^‐MTX, rituximab lenalidomide and methotrexate; R‐MA, rituximab methotrexate and cytarabine.

### 3.4 Safety and QoL assessment

In the present study, the safety profile assessment was relatively limited, and not all adverse effects were thoroughly documented in the medical records. However, the reasons for LEN dose reduction and withdrawal were documented: One patient prematurely discontinued LEN owing to drug‐related severe pneumonia, and 10 had their LEN dose reduced: eight owing to persistent grade 3–4 neutropenia, one owing to grade 3 vomiting and anorexia, and one owing to grade 3 rash.

**FIGURE 3:**
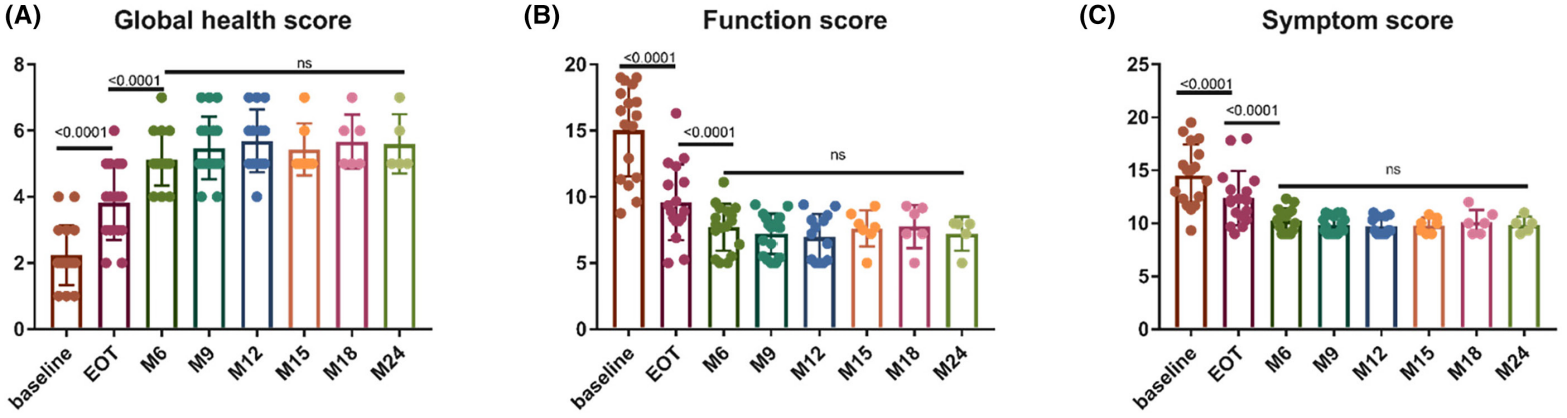
Dynamic change in EORTC QLQ‐C30 during treatment. (A) QLQ‐C30 global health score. (B) QLQ‐C30 function scores. (C) QLQ‐C30 symptom score. EORTC QLQ‐C30, European Organization for Research and Treatment of Cancer QLQ‐C30.

During the LEN maintenance phase, QoL was evaluated in 17 patients enrolled in the prospective REMLA study. The average global health score, as measured by the EORTC QLQ‐C30, exhibited a significant increase after induction therapy (from baseline 2.23 ± 0.88 to EOT 3.82 ± 1.10, *p* < 0.001; paired *t*‐test) and remained consistent throughout the maintenance phase (*p* = 0.51, analysis of variance), suggesting that patients remained in remission and that LEN maintenance minimally impacted the QoL. The mean function and symptom scores of QoL‐C30 exhibited a similar trend with the global health score and remained stable during the maintenance phase (Table S2 and Figure 3A–C).

## 4 DISCUSSION

To our knowledge, this study represents the first retrospective analysis examining the effectiveness of lenalidomide maintenance therapy in patients with PCNSL. However, our findings reveal that LEN maintenance did not demonstrate a significant advancement in PFS or OS in this particular series of cases.

Furthermore, our analysis revealed a crossover of the two survival curves at 30 months since the initiation of induction treatment (Figure 2A), which coincided with the termination of LEN maintenance. Prior to this time point, the LEN maintenance group exhibited a more favorable PFS curve, indicating that continuous LEN therapy may play a pivotal role in disease control. However, the necessity of extending the maintenance duration beyond 2 years warrants further evaluation. It is worth considering the findings of the study by Vu et al., wherein the majority of tumor relapse events occurred within the first year. In a multicenter study involving 256 relapsing/refractory patients in France, only 42 (16.4%) relapse events were reported to occur beyond the 12‐month mark.

The confirmation of a PFS benefit from LEN maintenance therapy through a comparison between LEN‐treated and nontreated patients was failed, and did not align with our initial hypothesis. One possible factor contributing to this inconsistency is the potential influence of WBRT consolidation, as a higher percentage of patients in the observation group underwent this treatment compared to the LEN group (33.3% vs. 6.0%, *p* = 0.004). However, approaching the issue from a different angle, the similar PFS observed in the LEN without WBRT and WBRT‐only groups (Figure 2C) suggests a comparable efficacy between LEN and WBRT. ASCT consolidation has been shown to prolong PFS and may potentially interfere with the outcomes of lenalidomide maintenance. However, in this study, both the observation group and the lenalidomide maintenance group had two patients each who underwent ASCT consolidation, and the impact on PFS was not as pronounced as that of WBRT. Furthermore, our analysis revealed a crossover of the two survival curves at 30 months since the initiation of induction treatment (Figure 2A), which coincided with the termination of LEN maintenance. Prior to this time point, the LEN maintenance group exhibited a more favorable PFS curve, indicating that continuous LEN therapy may play a pivotal role in disease control. However, the necessity of extending the maintenance duration beyond 2 years warrants further evaluation.

Considering the study by Vu et al., the majority of tumor relapse events occurred within the first year. In a multicenter study assessing 256 relapsing/refractory patients in France, only 42 (16.4%) relapse events occurred beyond the 12‐month mark.^14^ Therefore, a duration of 2 years for LEN maintenance appears to be a reasonable choice. Additionally, it is important to note that in this retrospective study, patients were enrolled over a period of almost two decades, and three different induction regimens were utilized. These variations may have influenced the outcomes. However, two real‐world retrospective studies conducted in the United States and Australia reported no significant association between the intensity and type of high‐dose methotrexate (HD‐MTX) based induction regimens and PFS and OS.^15^, ^16^

Despite the lack of difference in PFS between the maintenance and observation groups, there was a slight increase in OS within the maintenance group (*p* = 0.091). The discrepancy in benefits between PFS and OS could be mainly attributed to the different recruitment periods. The maintenance group was initiated from 2015 onwards during which time there may have been advancements in salvage and supportive treatments that contributed to the prolongation of OS.

In the subgroup analysis, LEN maintenance was significantly associated with prolonged PFS and OS in patients who achieved PR after induction therapy. This noteworthy finding suggests that LEN maintenance may serve as a viable option for patients who fail to achieve CR and are ineligible for intensive consolidation. Moreover, this finding differs from that of the REMARC study, which suggested that both patients who achieved CR and those who achieved PR shared the same PFS benefit.^10^ This difference could be due to the lack of conversion from PR to CR in the REMARC study.

Although LEN effectively penetrates the BBB at ≥15 mg, low‐dose treatment can enhance proliferation and suppress stimulated T‐cell apoptosis. This immunomodulatory effect may contribute to prolonged disease control in older patients with PCNSL.^11^, ^12^ In this study, we opted for a dosage of 25 mg, and the younger age and better tolerance of our patients were the main concerns.

The limitations of the present study need to be addressed. The lack of a safety profile was the major deficiency in this study. We only recorded the frequency, reasons for premature treatment discontinuation, and dose reductions. However, we analyzed the QoL from the prospective study (REMLA study) during maintenance to evaluate the impact on patients' lives. This retrospective study shared similar biases with other retrospective studies, such as the bias of baseline characteristics and treatment regimens. The limited number of cases was another important limitation. PCNSL is a rare subtype of DLBCL, and conducting large‐scale studies remains a considerable challenge. Significant progress has been made in genetic research in PCNSL patients recently, and these findings hold promising potential as therapeutic targets. However, an exploration of the relationship between gene mutations and the effectiveness of lenalidomide maintenance therapy has not been conducted. It should be acknowledged that this is a direction for future research.

In conclusion, the results of this retrospective study indicate that LEN maintenance following the induction therapy was well tolerated in younger patients with PCNSL. However, it did not demonstrated a significantly extention of PFS or OS, except for those patients who achieve PR. It is worthy noting that this study is the first of its kind to compare the efficacy of immunomodulatory agents as maintenance therapies for patients with PCNSL.

## AUTHOR CONTRIBUTIONS

**Yan Zhang:** Data curation (equal); funding acquisition (lead); methodology (lead); software (equal); writing – original draft (lead); writing – review and editing (lead). **Wei Wang:** Data curation (supporting); investigation (supporting); resources (equal). **Danqing Zhao:** Data curation (supporting); investigation (supporting); resources (supporting). **Wei Chong:** Data curation (supporting); investigation (supporting); resources (supporting); writing – original draft (supporting). **Chao Chen:** Data curation (equal); writing – original draft (equal). **Wei Zhang:** Conceptualization (equal); investigation (equal); writing – review and editing (equal). **Daobin Zhou:** Conceptualization (equal); supervision (equal); writing – review and editing (equal).

## FUNDING INFORMATION

This work was supported by the Capital's Funds for Health Improvement and Research (grant number: 2024‐2‐4011), National High‐Level Hospital Clinical Research Funding (grant number: 2022‐PUMCH‐B‐029), and CAMS Innovation Fund for Medical Sciences (CIFMS) (grant number: 2021‐I2M‐C&T‐B‐005).

## CONFLICT OF INTEREST STATEMENT

The authors have no relevant financial or nonfinancial interests to disclose.

## ETHICS STATEMENT

This study was performed in line with the principles of the Declaration of Helsinki. Approval was granted by the Peking Union Medical College Hospital Institutional Review Board (No. I‐23PJ920).

## CONSENT TO PARTICIPATE

Informed consent was waiver based on Peking Union Medical College Hospital Institutional Review Board.

## DECLARATION OF GENERATIVE AI IN SCIENTIFIC WRITING

No AI or AI‐assisted technologies were used in the writing process.

## Supporting information


Appendix S1.

## Data Availability

The datasets generated during and/or analyzed during the current study are available from the corresponding author on reasonable request.
