# Supplementary material for: The role of upfront lenalidomide maintenance for primary central nervous system lymphoma following first‐line methotrexate treatment: A retrospective study
Source: Cancer Med. 2024 May 13;13(9):e7193. doi: 10.1002/cam4.7193 (PMC11089434; doi:10.1002/cam4.7193)
Supplement: Supplementary file 1 — Appendix S1. [file CAM4-13-e7193-s001.pptx]

## Slide 1
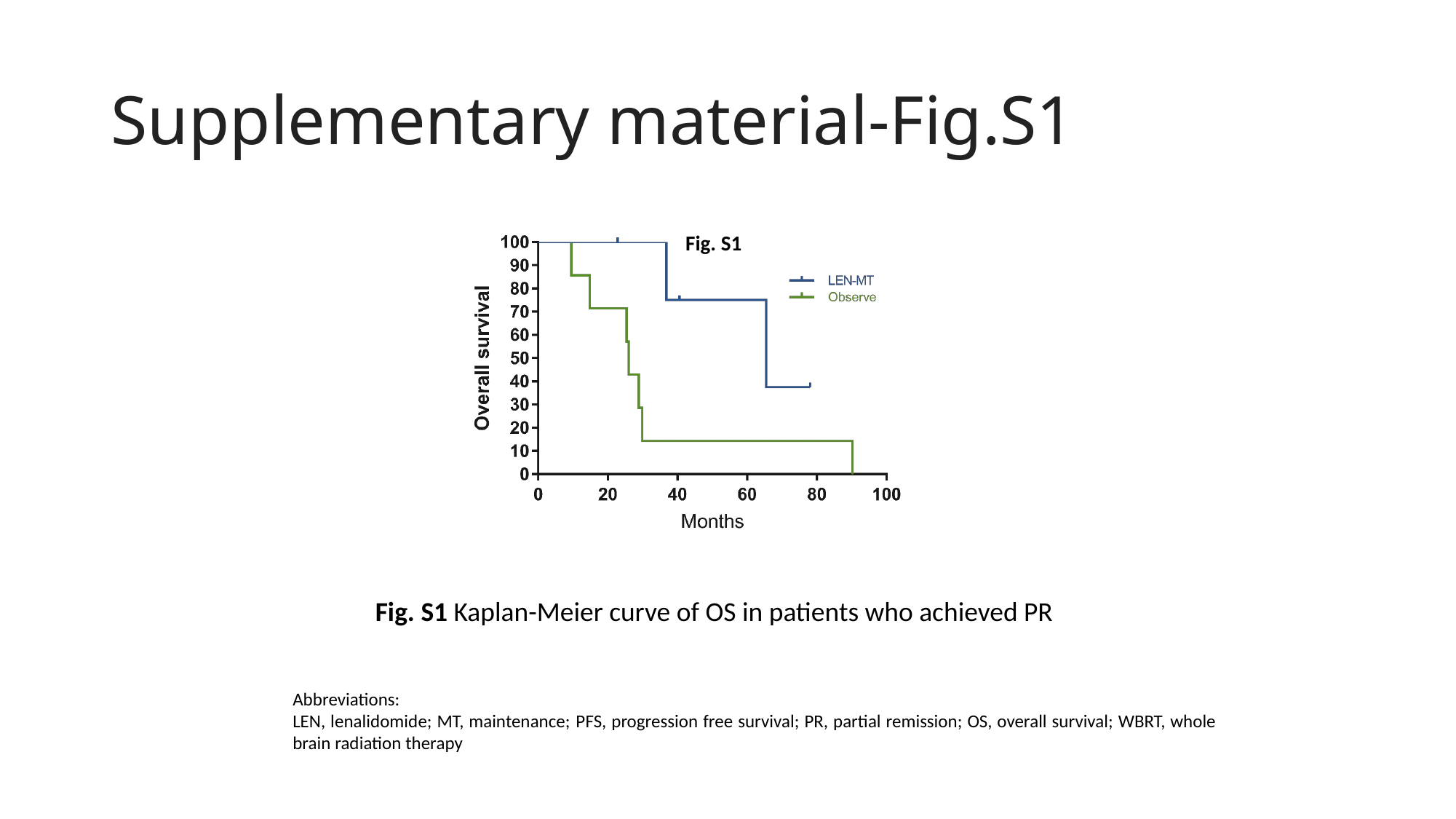

# Supplementary material-Fig.S1
Fig. S1
Fig. S1 Kaplan-Meier curve of OS in patients who achieved PR
Abbreviations:
LEN, lenalidomide; MT, maintenance; PFS, progression free survival; PR, partial remission; OS, overall survival; WBRT, whole brain radiation therapy

## Slide 2
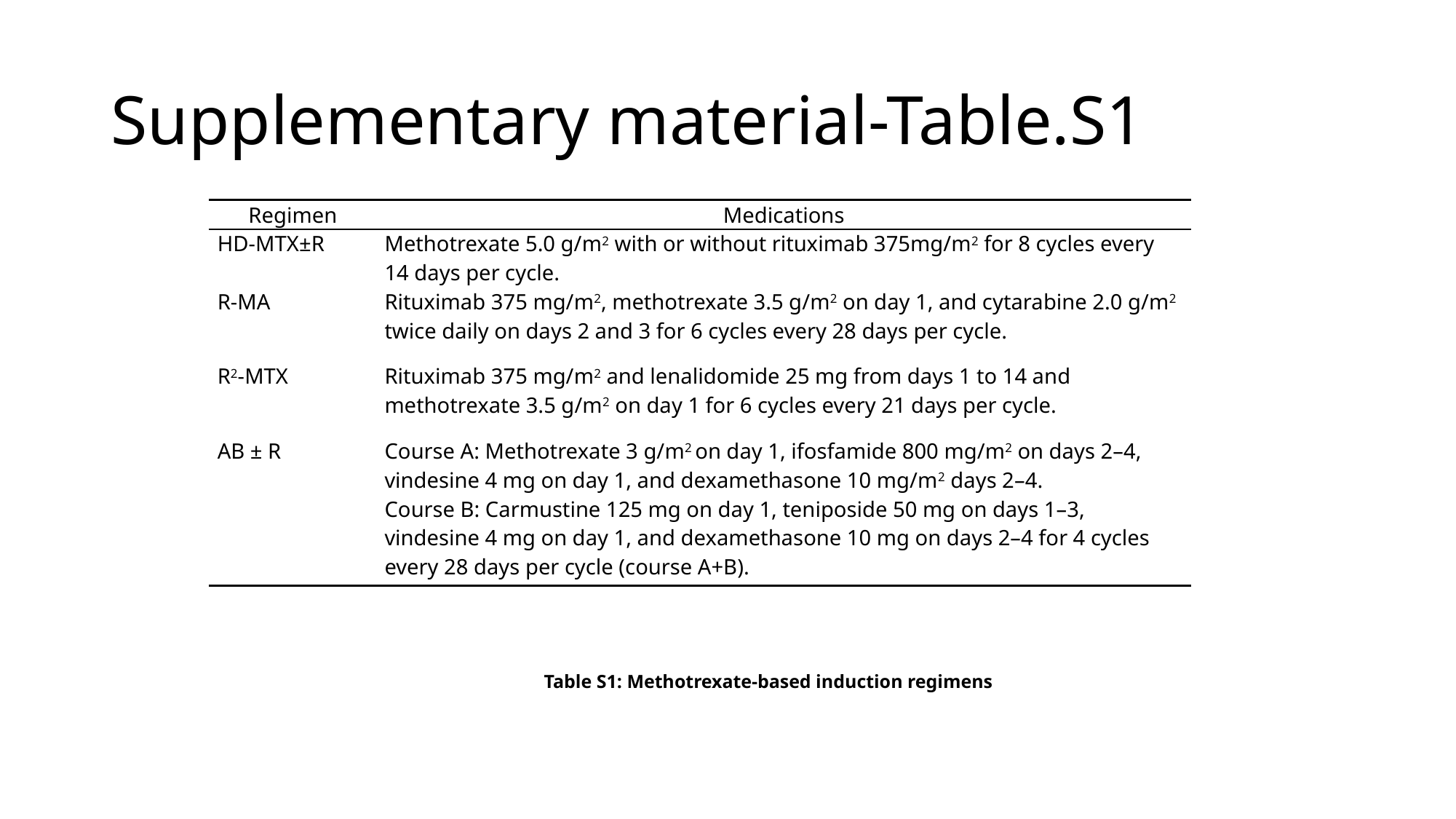

# Supplementary material-Table.S1
| Regimen | Medications |
| --- | --- |
| HD-MTX±R | Methotrexate 5.0 g/m2 with or without rituximab 375mg/m2 for 8 cycles every 14 days per cycle. |
| R-MA | Rituximab 375 mg/m2, methotrexate 3.5 g/m2 on day 1, and cytarabine 2.0 g/m2 twice daily on days 2 and 3 for 6 cycles every 28 days per cycle. |
| R2-MTX | Rituximab 375 mg/m2 and lenalidomide 25 mg from days 1 to 14 and methotrexate 3.5 g/m2 on day 1 for 6 cycles every 21 days per cycle. |
| AB ± R | Course A: Methotrexate 3 g/m2 on day 1, ifosfamide 800 mg/m2 on days 2–4, vindesine 4 mg on day 1, and dexamethasone 10 mg/m2 days 2–4. Course B: Carmustine 125 mg on day 1, teniposide 50 mg on days 1–3, vindesine 4 mg on day 1, and dexamethasone 10 mg on days 2–4 for 4 cycles every 28 days per cycle (course A+B). |
Table S1: Methotrexate-based induction regimens

## Slide 3
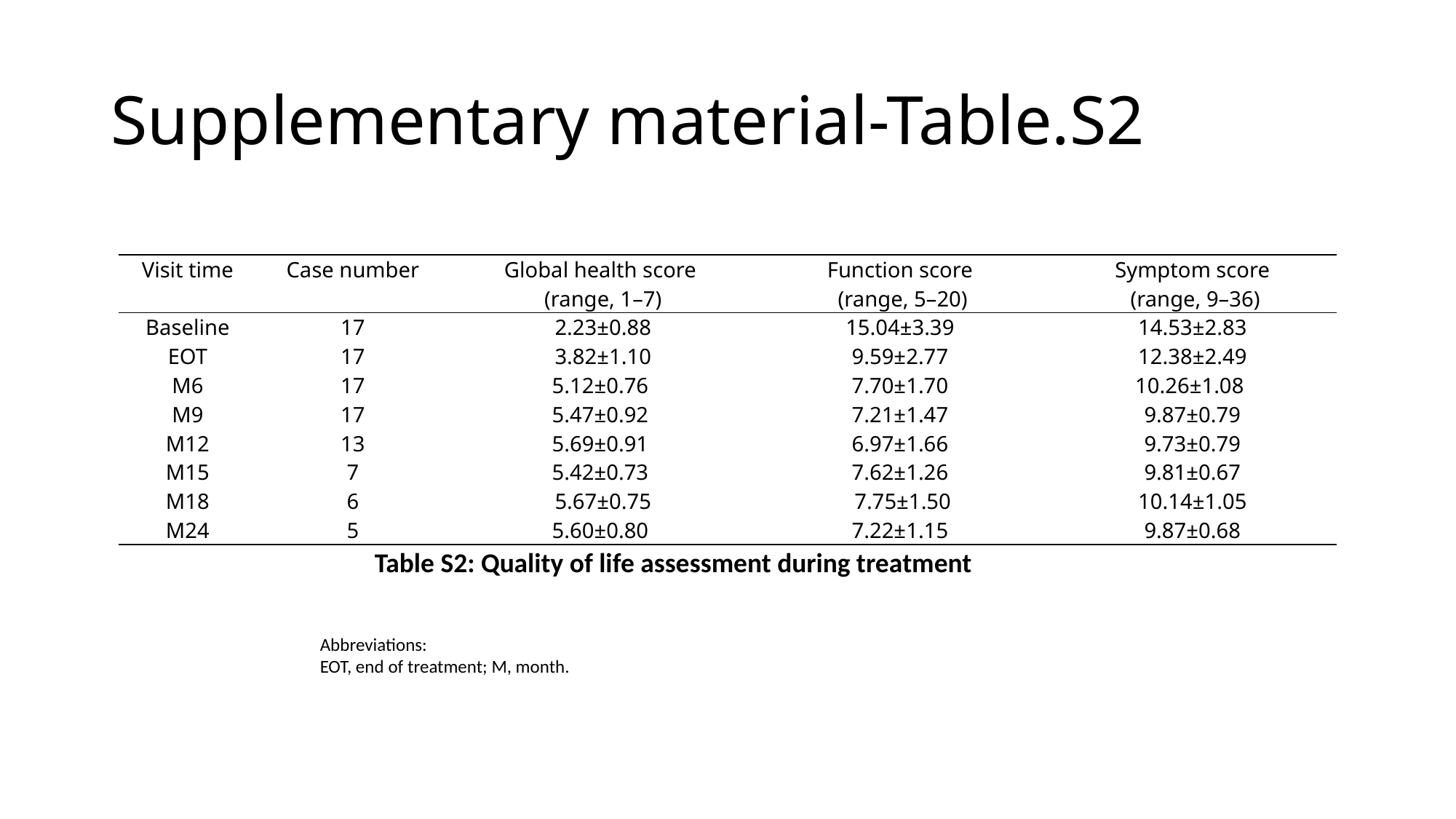

# Supplementary material-Table.S2
| Visit time | Case number | Global health score (range, 1–7) | Function score (range, 5–20) | Symptom score (range, 9–36) |
| --- | --- | --- | --- | --- |
| Baseline | 17 | 2.23±0.88 | 15.04±3.39 | 14.53±2.83 |
| EOT | 17 | 3.82±1.10 | 9.59±2.77 | 12.38±2.49 |
| M6 | 17 | 5.12±0.76 | 7.70±1.70 | 10.26±1.08 |
| M9 | 17 | 5.47±0.92 | 7.21±1.47 | 9.87±0.79 |
| M12 | 13 | 5.69±0.91 | 6.97±1.66 | 9.73±0.79 |
| M15 | 7 | 5.42±0.73 | 7.62±1.26 | 9.81±0.67 |
| M18 | 6 | 5.67±0.75 | 7.75±1.50 | 10.14±1.05 |
| M24 | 5 | 5.60±0.80 | 7.22±1.15 | 9.87±0.68 |
Table S2: Quality of life assessment during treatment
Abbreviations:
EOT, end of treatment; M, month.
